# Impact of cognitive reserve on dance intervention-induced changes in brain plasticity

**DOI:** 10.1038/s41598-021-97323-2

**Published:** 2021-09-17

**Authors:** Kristína Mitterová, Patrícia Klobušiaková, Alžběta Šejnoha Minsterová, Sylvie Kropáčová, Zuzana Balážová, Jaroslav Točík, Pavlína Vaculíková, Alena Skotáková, Roman Grmela, Irena Rektorová

**Affiliations:** 1 Applied Neuroscience Research Group, Central European Institute of Technology, Masaryk University, Kamenice 5, 62500 Brno, Czech Republic; 2 Faculty of Medicine, Masaryk University, Kamenice 5, 62500 Brno, Czech Republic; 3 Surgeon General Office of the Slovak Armed Forces, Ul. generála Miloša Vesela 21, 03401 Ružomberok, Slovak Republic; 4 Department of Gymnastics and Combatives, Faculty of Sports Studies, Masaryk University, Kamenice 5, 62500 Brno, Czech Republic; 5 Department of Health Promotion, Faculty of Sports Studies, Masaryk University, Kamenice 5, 62500 Brno, Czech Republic; 6 First Department of Neurology, Faculty of Medicine, Masaryk University and St. Anne’s University Hospital, Pekařská 664/53, 65691 Brno, Czech Republic

**Keywords:** Cognitive ageing, Neural ageing, Regeneration and repair in the nervous system, Neurological disorders

## Abstract

Dance is a complex sensorimotor activity with positive effects on physical fitness, cognition, and brain plasticity in the aging population. We explored whether individual levels of cognitive reserve (CR) proxied by education moderate dance intervention (DI)-induced plasticity assessed by resting-state functional connectivity (rs-FC) changes of the sensorimotor network (SMN), and between the dorsal attention network (DAN) and anterior default mode network (aDMN). Our cohort consisted of 99 subjects, randomly assigned to either a DI group who underwent a 6-month intervention (n = 49, M_age_ = 69.02 ± 5.40) or a control group (n = 50, M_age_ = 69.37 ± 6.10). Moderation analyses revealed that CR moderated DI-induced increase of the SMN rs-FC with significant changes observed in participants with ≥ 15 years of education (b = 0.05, t(62) = 3.17, p = 0.002). Only DI alone was a significant predictor of the DAN–aDMN crosstalk change (b = 0.06, t(64) = 2.16, p = 0.035). The rs-FC increase in the SMN was correlated with an improved physical fitness measure, and changes in the DAN–aDMN connectivity were linked to better performance on figural fluency. Consistent with the passive CR hypothesis, we observed that CR correlated only with baseline behavioral scores, not their change.

## Introduction

Dance is a complex sensorimotor activity that involves learning new motor skills, utilizes attentional action observation and imitation, and integrates sensory, motor, and cognitive demands^1^ that bestow rehabilitating effects even on an aging brain. Considerable experimental research on dance intervention (DI) in the elderly has shown compelling benefits in postural and gait parameters^2^, physical fitness^3^, and cognition in the memory^4,5^, attention^2,6^, and psychosocial domains^7^. A recent meta-analysis supported the rich benefits of DI on global cognition and memory, but not on the inhibition and task-switching aspects of executive functioning^8^. Overall, DI-induced behavioral benefits are key in preserving mobility and independence in older age^9^ and the importance of studying them stems particularly from the low efficacy of current pharmacological treatment for dementia patients^10^.

Our previous research of an optimized, structured 6-month-long dance intervention (DI) on non-demented seniors demonstrated its positive effects in comparison with “life activities as usual” (LAU) on the performance of the 8-Foot Up-and-Go (8UG) and the 30-Second Chair Stand (30CS) tests^11^ which target dynamic balance, agility, lower body strength, and physical endurance^9^; and of the Five Point Test (FPT)^12,13^, which assesses figural fluency, i.e. the ability of executive functions to provide information about divergent reasoning, divided attention, planning, and mental flexibility^14^. Interestingly, despite the fact that figural fluency is known to decline in the healthy elderly and in patients with Alzheimer’s disease (AD)^15^, the observed improvements were independent of hippocampal volumes^12^. This finding indicates an individual capacity to recruit additional neural resources in order to meet the demands of the intervention. To test this hypothesis, the current work aims at studying neural changes associated with the described behavioral improvements in terms of neural compensation. This accords well with Lövdén and colleagues, who postulated that any acquisition of new skills (dancing, in our case) requires changes in neuronal connections provided by the brain’s capacity for plasticity termed as a cognitive reserve (CR)^16^. CR is usually proxied by lifetime exposure to cognitively enriching activities^17,18^ with “years of education” being the most common proxy of CR^19^. The CR bridges the disjunction between brain pathology load and preserved cognition, and promotes the optimization and recruitment of brain networks^20^. This is achieved by various mechanisms, among which the increased engagement and connectivity of large-scale brain networks plays an important role^18,21^. Several authors attempted to examine CR-related changes at the level of resting-state brain networks with inconclusive results^22^. Others showed that the level of CR has an impact on the association between the magnitude of resting-state connectivity and cognitive outcomes. For example, lower anti-correlation between an anterior (frontal) part of the default mode network (aDMN) and the dorsal attention network (DAN) was linked with decreased memory performance in those amnestic mild cognitive impairment (aMCI) patients who had low CR (proxied by education and IQ), but not in those with high CR levels^23^. In other words, higher levels of CR alleviated the impact of disrupted inter-network crosstalk on cognition. A rich body of research also supported CR effects in healthy elderly subjects, particularly on preserving inhibition, flexibility, working memory, and visuo-perceptive functions^24^, but also on alleviating the negative impact of white matter lesions on motor functions^25^.

In the current study, we aimed to explore the extent to which the individual level of CR moderates the intervention-induced ability to recruit behaviorally-relevant neural resources as assessed by resting-state fMRI. We were specifically interested in the resting-state functional connectivity (rs-FC) changes of the sensorimotor network (SMN) and in the inter-network connectivity changes between the DAN and the aDMN, with the ventromedial prefrontal cortex (vmPFC) as a representative region. To provide a comprehensive picture, changes of other large-scale network pairings^26^ are analyzed in an exploratory manner. The rationale behind the first network of interest was the fact that the SMN connectivity increases in response to motor training^27,28^. The SMN is a set of highly interconnected somatosensory, primary motor, and premotor regions that coordinate action and operate in a hierarchical fashion to translate visual and rule-based information into appropriate motor responses^29^. Regarding the DAN–aDMN, activity within the aDMN node in the anterior cingulate cortex encompassed in the vmPFC has been associated with the modulation of reaction times^30^, as well conflict resolution and responses within the constraints of a task, such as during fluency tasks^31^. The DAN consists particularly of the bilateral superior parietal lobules/ intraparietal sulci and the frontal eye fields, and since it contributes to the formation of task rules and goals by top-down orienting, it is engaged especially during externally directed tasks^32^. Conversely, the DMN is a set of highly interconnected brain regions active when the mind is not engaged in specific behavioral tasks and suppressed (deactivated) during goal-directed behavior with focused attention^33^. The anterior part of the DMN is particularly connected to the parietal regions of the DAN, and it has been associated with attention and memory functions^34^. It has been well documented that the magnitude of the rs-FC between the task-positive DAN and the task-negative DMN plays a central regulating role within functional networks underlying cognition^35^. Patterns of the DAN–DMN connectivity reflect cognitive control efficiency and working memory^33^ as well as episodic memory performance^23,36^. Finally, we used the graph metrics of global efficiency and modularity to assess whether CR or the DI modulate general age-related network changes^37^.

Taken together, there is a sufficient evidence that CR buffers the impact of normal or pathological aging on cognition. Individuals with high CR are more capable of drawing brain plasticity changes on the scale of rs-FC^22^, and hence meet demands of an intervention. Moreover, dancing requires participants to learn complex motor sequences, and thus may interact with previous levels of education. The abovementioned studies have motivated our aim to study the moderating effects of CR in the context of intervention-induced benefits. More specifically, we directly probe the effect of DI on rs-FC changes at different levels of education as a proxy of CR.

## Results

### Baseline measures and their changes: comparisons of the groups

No significant baseline differences were found between the DI (n = 49) and the LAU (n = 50) groups in sex (X^2^(1) = 2.77; p = 0.10) and MCI (n = 21; X^2^(1) = 1.24; p = 0.27) incidence, demographic data, screening of general cognition (MoCA), behavioral tests of interest, or in the connectivity of networks of interest. Note that 68 participants (DI: 36, LAU: 32) had complete fMRI data. All relevant baseline measurements and their changes are depicted in Table 1. As reported in our previous works^11,12^, changes in the FPT, 8-Foot Up-and-Go, and 30-Second Chair Stand test scores significantly differed between the DI and the LAU groups. Similarly, the change in the rs-FC of DAN–aDMN significantly differed between both groups. For baseline and follow-up comparisons of cognitive tests that were not included in the present text, see Kropacova et al.^12^; no significant baseline differences were found. Baseline and changes were compared separately for the MCI vs HC and DI vs LAU groups using Mann Whitney U test (see “Supplementary Materials”, Tables S3a and S3b). No variables of interest were driven by the MCI group.

**Table 1:** Baseline and follow-up demographic comparison of two experimental groups based on Mann–Whitney U test for independent samples.

Variables	Baseline variables				Change of variables (time_2_-time_1_)			
	DI	LAU	*t(df)*	*p*	DI	LAU	*t(df)*	*p*
	M ± SD	M ± SD			M ± SD	M ± SD		
Age	69.10 ± 5.43	68.37 ± 6.10	− 0.63 (97)	0.53	–	–	–	–
Education	14.53 ± 2.58	14.80 ± 3.10	0.47 (97)	0.64	–	–	–	–
MoCA	26.92 ± 2.85	26.06 ± 2.71	− 1.53 (97)	0.13	− 0.20 ± 2.42	0.66 ± 2.37	1.80 (97)	0.075
FPT	28.39 ± 8.36	31.12 ± 9.48	1.52 (97)	0.13	0.54 ± 0.92	− 0.02 ± 1.09	− 2.81 (97)	0.006
8UG	5.08 ± 1.21	5.23 ± 1.28	0.60 (96)	0.55	− 0.31 ± 0.76	0.31 ± 1.22	2.98 (93)	0.004
30CS	15.85 ± 3.51	16.43 ± 4.53	0.70 (95)	0.49	1.58 ± 2.62	0.31 ± 2.36	− 2.46 (91)	0.02
SMN	0.54 ± 0.10	0.58 ± 0.18	1.17 (48.25)	0.25	0.04 ± 0.16	− 0.01 ± 0.15	− 1.27 (66)	0.21
DAN–aDMN	0.06 ± 0.08	0.08 ± 0.12	1.03 (53.51)	0.31	0.02 ± 0.10	− 0.03 ± 0.11	− 2.23 (66)	0.03

*DI* dance-intervention group, *LAU* life-as-usual group, *Age* and *Education* in years, *MoCA* Montreal Cognitive Assessment score, *FPT* Five-Point Test score, *8UG* 8-Foot Up-and-Go score in seconds, *30SC* 30-Second Chair Stand test score, *SMN* internetwork connectivity of the sensorimotor network, *DAN–aDMN* connectivity between the dorsal attention network and the anterior default mode network, *M* mean, *SD* standard deviation.

### Correlation analyses

Table 2 display one-tailed Pearson correlations of networks and tests of interest and education; bold values are significant correlations after adjusting for age and sex. Baseline fitness scores were mutually correlated, as well as the FPT with the 8UG task and with the 30SC test. The baseline rs-FC of the SMN and of the DAN–aDMN were not correlated with any of the baseline behavioral tests of interest. Finally, the improvement in the 8UG correlated with increased connectivity within the SMN and improvement in the FPT correlated with the rs-FC change between the DAN–aDMN, but the latter correlation reached significance only after adjusting for age (r = 0.21, p = 0.050).

**Table 2:** Pearson correlations of behavioral and rs-FC outcomes, and CR proxy.

	CR	MoCA	FPT	30SC	8UG	SMN	aDMN–DAN	DMN–DAN
CR	1							
MoCA	**0.22**^b^	1						
FPT	0.18^b^	0.07	1					
30SC	**0.37**^a^	0.12	0.21^b^	1				
8UG	− 0.22^b^	− 0.17	− 0.24^b^	− **0.68**^a^	1			
SMN	0.01	0.02	− 0.02	0.11	0.08	1		
aDMN–DAN	− 0.03	− 0.04	0.10	0.22^b^	− 0.13	0.08	1	
DMN–DAN	− 0.08	0.10	0.17	0.21^b^	− 0.14	**0.29**^a^	**0.76**^a^	1

	CR	Δ MoCA	Δ FPT	Δ 30SC	Δ 8UG	Δ SMN	Δ aDMN–DAN	Δ DMN–DAN
CR	1							
Δ MoCA	**0.22**^b^	1						
Δ FPT	0.00	− **0.23**^b^	1					
Δ 30SC	0.12	− 0.09	0.01	1				
Δ 8UG	− 0.11	− 0.07	− 0.01	− **0.40**^a^	1			
Δ SMN	0.02	0.09	− 0.02	− 0.07	− **0.21**^b^	1		
Δ aDMN–DAN	0.03	− **0.13**	**0.19**	− 0.01	− 0.18	0.01	1	
Δ DMN–DAN	0.08	0.03	0.18	0.05	− 0.07	**0.22**^b^	**0.70**^a^	1

Bold values suggest significant partial correlations after adjusting for age and sex.

∆-change time_2_ − time_1_, *CR* cognitive reserve, *MoCA *Montreal Cognitive Assessment test, *FPT* Five-Point Test, *8UG* 8-Foot Up-and-Go score in seconds, *30SC* 30-Second Chair Stand test score, *SMN* internetwork connectivity of the sensorimotor network, *DAN–(a)DMN* connectivity between the dorsal attention network and the (anterior) default mode network.

^a^Correlation is significant at the 0.01 level (1-tailed).

^b^Correlation is significant at the 0.05 level (1-tailed).

Education was positively related to all baseline behavioral scores: FPT, the 8-Foot Up-and-Go, the 30-Second Chair Stand test, and the MoCA, but only to the change of the MoCA score. Consistent with the moderation model (see below), education correlated only with the rs-FC change within the SMN in the DI group (r = 0.41, p = 0.007) but not with its baseline; it did not correlate with either the baseline rs-FC between DAN–aDMN nor with the between-network rs-FC change.

Exploratory Pearson correlations between all cognitive tests administered in the study and education are reported in the “Supplementary Materials” (Tables S4a, S4b).

### Moderation analyses

**Figure 1:**
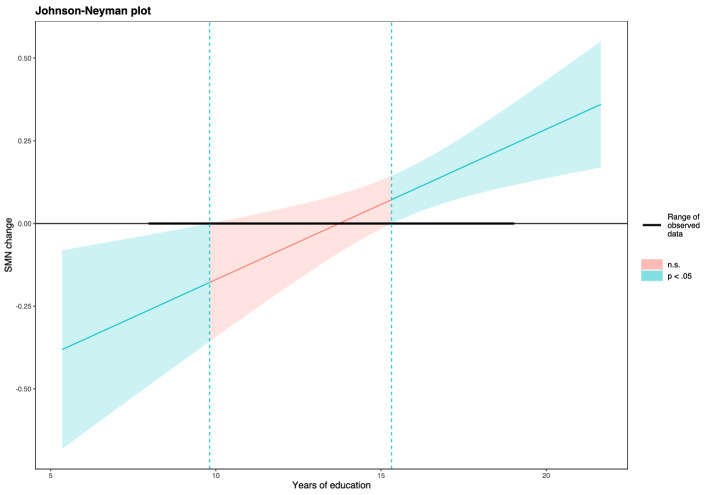
Johnson–Neyman plot depicting conditional effects of dance intervention on change within the sensorimotor network (*SMN change*) at values of the moderator cognitive reserve (*years of education*). The plot shows the range of observed data (bold line) and the zone of significance.

The moderation analysis of rs-FC change within the SMN revealed that education moderates the effect of DI on the network change, i.e. the effect of dance depended on years of education. No main effect was significant in this model and only the interaction effect was observed. Upon closer inspection of the Johnson–Neyman zone of significance (Fig. 1), we observed a significant positive effect in higher values of CR (cut-off 15.34 years of education; operationalized as W = 0.423; 44.12% of cases), which diminished in moderate values of education. Conversely, as education decreased (cut-off 9.80 years of education; W = − 4.885; 4.41% of cases) the relationship between DI and rs-FC change was significantly negative.

As for the second hypothesized DAN–aDMN between-network rs-FC, the moderation analysis revealed that changes were dependent on the main effect of program; the effects of the covariates, education or the interaction were not significant. The regression coefficients and t values that emerged from the moderation analyses of rs-FC changes are presented in Table 3.

**Table 3:** Linear model of predictors of change in SMN connectivity (R^2^ = 0.166; F(5,62) = 3.065; p = 0.016) and DAN–aDMN connectivity (R^2^ = 0.088; F(5,62) = 1.023; p = 0.412).

Model terms	B [95% CI]	SE B (HC4)	t	p
**SMN change**				
Constant	− 0.071 [− 0.643, 0.500]	0.286	− 0.250	0.803
Program	0.053 [− 0.021, 0.127]	0.037	1.430	0.158
Cognitive reserve (centered)	− 0.016 [− 0.035, 0.003]	0.010	− 1.709	0.093
Moderator CR*program	0.045 [0.017, 0.074]	0.014	3.169	0.002
Age	0.001 [− 0.007, 0.009]	0.004	0.285	0.777
Sex	− 0.016 [− 0.100, 0.069]	0.042	− 0.375	0.709
**DAN–aDMN change**				
Constant	− 0.064 [− 0.353, 0.225]	0.145	− 0.441	0.661
Program	0.062 [0.005, 0.119]	0.029	2.160	0.035
Cognitive reserve (centered)	− 0.001 [− 0.018, 0.015]	0.008	− 0.182	0.856
Moderator CR*program	0.008 [− 0.014, 0.029]	0.011	0.717	0.476
Age	0.001 [− 0.003, 0.005]	0.002	0.315	0.754
Sex	− 0.021 [− 0.102, 0.059]	0.040	− 0.523	0.603

Results are unstandardized beta coefficients with 95% confidence intervals from moderation models estimating the association of program, *CR* (years of education), and their interaction with rs-FC change. Note: only continuous variables that contributed to the outcomes were centered. Cribari-Neto model was used for standard error of variance and F-statistics. Variable program coded as *LAU*: 0, *DI*: 1; *SMN* inter-network rs-FC of the sensorimotor network; *DAN–aDMN* between-network rs-FC of dorsal attention network and anterior part of the default mode network.

The exploratory analyses of other networks are presented in detail in the “Supplementary Materials” (Table S5). DI (without the contribution of education or the covariates) was a significant positive predictor of the (whole) DAN–DMN between-network connectivity increase t(df) = 2.17, p = 0.034. The models predicting change between the DMN and the FPCN, VN, and SMN were not significant.

Additionally, we performed moderation analyses on the global efficiency and modularity. These exploratory analyses were carried out in a similar fashion, and a detailed report can be found in the “Supplementary Materials” (Table S6). In brief, global efficiency increased in response to the DI (t(62) = 2.20, p = 0.032) invariantly on education or the covariates. We did not observe significant changes in the global modularity.

## Discussion

Dance is a joyful complex activity combining physical exercise with cognitive, social, musical, and artistic stimulation^38^. The benefits of dance intervention stem from the recruitment of higher-order cognitive functions that require enhanced engagement and coordination of large-scale brain networks^39^. This study followed up on our previous findings showing that DI elicits distinct motor^11^ and cognitive improvements^12^; it focused on the moderating effects of CR proxied by education on the DI-induced rs-FC changes involving the large-scale brain networks, namely the SMN and the DAN–aDMN.

We observed that the rs-FC increase of DAN–aDMN was dependent on the DI, while the changes in the SMN intra-network connectivity depended on the interaction between the DI and education. In other words, the follow-up changes within the SMN network were not significant across the whole DI group but only in those with higher education. This result is discussed in more detail in the context of CR moderating effects in the text below.

All the baseline behavioral (fitness and cognitive) scores as well as their follow-up improvements were mutually correlated, sharing approximately 10% of their variance. This variance can represent a shared component of psychomotor speed, which declines throughout the lifespan^40^.

Regarding our neuroimaging results, previous literature showed that dance practice may modify brain plasticity as evaluated by structural MRI^13,41^, but little is known about the rs-FC changes induced by the DI. By comparing professional and naïve dancers, Burzynska et al.^38^ demonstrated differences between both groups in the engagement of the general motor learning network, including major nodes of the SMN, basal ganglia structures, and frontoparietal regions. The current study employed rs-fMRI and for the first time explored the CR moderation of brain plasticity changes resulting from the DI. Our major finding supported the significant CR moderation (proxied by education) of the DI effect on the rs-FC changes within the SMN. Specifically, the observed increase of the SMN rs-FC was dependent on ≥ 15 years of education (which equals the minimum of secondary education with graduation in the Czech schooling system). This effect dissolved in moderate levels and significantly reversed in low levels of education (≤ 10 years of education). Note that only 4.41% of the cases had such a low education level, and thus this latter result cannot be further interpreted. The observed DI-induced increase of rs-FC of the SMN is clinically relevant as it was associated with improved performance in dynamic balance and mobility, known to decline with aging^9^. The SMN is particularly engaged in motor learning and execution of specific motor actions^42^; although the SMN has not been assessed in the context of DI, studies on aerobic exercise interventions have consistently reported a reactive increase of rs-FC of the SMN in healthy^28^ and diseased subjects^43^, as well as significant differences in its structural connectivity among professional ballet dancers in comparison to a control group^44^. The relation between education level and motor network involvement may seem peculiar; nevertheless, associations between motor aspects and education levels have been demonstrated. For instance, in Parkinson’s disease patients (i.e. the typical patient group with a movement disorder) the CR (proxied by education) was inversely correlated with motor symptom severity despite greater reductions in dopamine levels^45,46^. In healthy elderly subjects, higher CR buffered the impact of white matter lesions on walking speed at baseline but did not influence the follow-up assessment^25^.

In contrast, the moderation model of the DAN–aDMN rs-FC changes revealed that even when accounting for age and sex, the DI alone, without CR contribution, is a significant predictor of its change. This was not specific just to the anterior node of the DMN, since we observed the same effects for the whole DAN–DMN. In contrast, we found that when accounting for age and sex, rs-FC change between the DAN–aDMN was significantly related to improvement in the figural fluency task; this was not true for the whole DAN–DMN. The DMN–DAN connectivity plays an important role in cognitive control and working memory^30,33^, which is significantly altered with aging^47^. Anthony and Lin^48^ speculate that individual hub seeds of the DMN, including the anterior cingulate region, underlie the core hub of *neural reserve* in the context of CR, while the DAN regions are rather related to *neural compensations* (i.e. engaged in brain maintenance to compensate for brain pathology). Therefore, our results are in line with the notion that by increasing the DAN–DMN crosstalk, dancing may facilitate neuroplasticity and the preservation of CR^49^. We also observed DI-induced increase of network efficiency, which has been linked to higher CR capacity in pathological aging^37,50^. A prospective 21-year study demonstrated that regular participation in dancing was the only physical activity among the 11 studied (e.g. bicycling, playing tennis or swimming) that was associated with a lower risk of dementia in an elderly cohort, presumably by increasing plasticity and CR^51^.

Interestingly, while higher levels of education proxy were related to better baseline behavioral scores, they were not correlated with their follow-up changes. Even though ours is the first study to observe such discrepancies resulting from an intervention, many longitudinal observational studies in healthy elderly subjects, and particularly those conducted on samples with a degenerative brain disease, found that CR (proxied by education, occupation, or premorbid IQ) was related to baseline behavioral outcomes, but not to their changes^52,53^. For instance, higher education among PD patients predicted lower incidence of high Hoehn–Yahr stage, better cognitive and motor baseline scores as estimated by MMSE and gait speed with UPDRS-III respectively, but not their annual progression of 6 years^54^. This phenomenon has been dubbed a passive reserve hypothesis and highlights the CR contribution to better cognitive and motor performance scores resulting from the persistence of differences that appear at younger ages, rather than from ongoing changes (e.g. lifestyle or pathology) that influence differential rates of cognitive decline^54^.

There are limitations to our study. We used a static proxy of CR which may not be reflective of the dynamic nature of the CR and its pathology-induced depletion^55^. Estimating dynamic CR using a latent or residual CR index^56,57^ in future research might deepen our understanding of its reactive nature. Besides, educational attainment is contaminated with socioeconomic factors, such as income, access to health care, gender, and healthy lifestyle habits. Second, by using a non-active “life activities as usual” control group, we were unable to control for other significant factors, e.g. the social aspect of this collective intervention. Finally, adaptive testing is a more sensitive approach to training or evaluation in uncovering post-intervention effects.

In conclusion, the protective effects of cognitive reserve in nondemented older adults have been suggested by several lines of research. We showed that an intensive 6-month DI can induce clinically-relevant changes in brain plasticity, physical fitness, and cognition, and importantly, that some of the brain plasticity changes depend on education, a proxy of CR, suggesting that higher capacity for plasticity applies to better intervention outcomes. Our study also demonstrated that the DAN–(a)DMN rs-FC, a potential neural representation of CR, can be modulated by DI. Future studies should employ multimodal comprehensive programs to benefit people across different CR levels^40^. Despite our clinically-relevant results, it is unknown whether short-term engagement in any set of activities is sufficient to elicit changes that last several months or even years after the intervention completion. Therefore, long-term behavioral outcomes of such interventions should be examined and long-term moderation effects of CR should be tested.

## Materials and methods

### Sample

A total of 99 community-dwelling, non-demented elderly (MCI and healthy) subjects completed the main study and were described in detail previously^10,11^. All subjects were over 60 years of age without any medical, neurological, or psychiatric disorders that may have an impact on cognition (such as major depression, drug and/or alcohol abuse), or would interfere with DI or with MRI scanning. The absence of dementia was assessed by a screening of cognitive decline (MoCA), the Functional Activities Questionnaire, and a detailed cognitive battery (see Table S1 in the “Supplementary Materials”). Subjects were randomized to a dance intervention group (DI) (N = 49) or a control (LAU) group (N = 50). For detailed information about the enrolment and randomization process, see Kropacova et al.^12^. Informed consent was obtained from each subject. The study was approved by the ethics committee of Masaryk University and the experiment was performed in accordance with relevant guidelines and regulations. Each subject underwent a neurological examination, detailed neuropsychological evaluation, MRI, and physical fitness examination prior to the program and six months after the program completed.

### Dance intervention

The DI program was designed and supervised by specialists from the Faculty of Sports Studies, Masaryk University, Brno, Czech Republic. The whole study lasted for three years with the yearly rotation of a group of 20 subjects. Each intervention took six months and included three training units (each of 60 min) per week. The DI program was supervised and conducted at a medium physical load intensity which was monitored each session using the Borg Rating of Perceived Exertion (RPE) scale, a user-friendly numerical scale that evaluates an individual’s subjective effort, physical exertion, and fatigue during exercise on a 15-point scale^58^. The DI sessions included folk, country, African, Greek, and tango dancing. The choreographies were divided into smaller blocks that were gradually taught in individual lessons and modified and developed over time into the final choreography. Only subjects who completed at least 60% of the DI program were included in the final cohort^12^. The real average completion of the DI program was 78.1%.

### Physical fitness examination

The effect of the DI was evaluated using two tests from the functional fitness assessment^9^. The 8-Foot Up-and-Go Test evaluates agility and dynamic balance. It measures the time (in seconds) required to get up from a seated position, walk an eight-foot distance, return to the chair, and sit down. Lower values indicate better performance. The 30-Second Chair Stand test evaluates lower body strength and physical endurance by measuring the number of repetitions of full stands from a chair in 30 s. Higher values indicate better performance. Scores on both tests improved in the DI group as compared to the LAU group^11^.

### Neuropsychological examination

Global cognition (MoCA), activities of daily living, and five cognitive domains were evaluated by complex neuropsychological testing (see Table S1 in the “Supplementary Materials”). The five domains included memory, attention, executive, visuospatial, and language domain. In the current study, we focus on the Five-Point Test (FPT) performance which significantly improved in the DI group as compared to the LAU group^12^.

### MRI examination

All subjects were scanned using the 3T Siemens Prisma MRI scanner (Siemens Corp., Erlangen, Germany) employing various sequences including the T1 anatomical and diffusion tensor imaging sequences^11,13^. For the purpose of this study, we used resting-state fMRI data, employing gradient-echo echo-planar imaging sequence (200 scans, 34 transversal slices, slice thickness = 3.5 mm, TR = 1990 ms, TE = 35 ms, FA = 70°, FOV = 192 mm, matrix size 64 × 64).

### fMRI data processing

**Figure 2:**
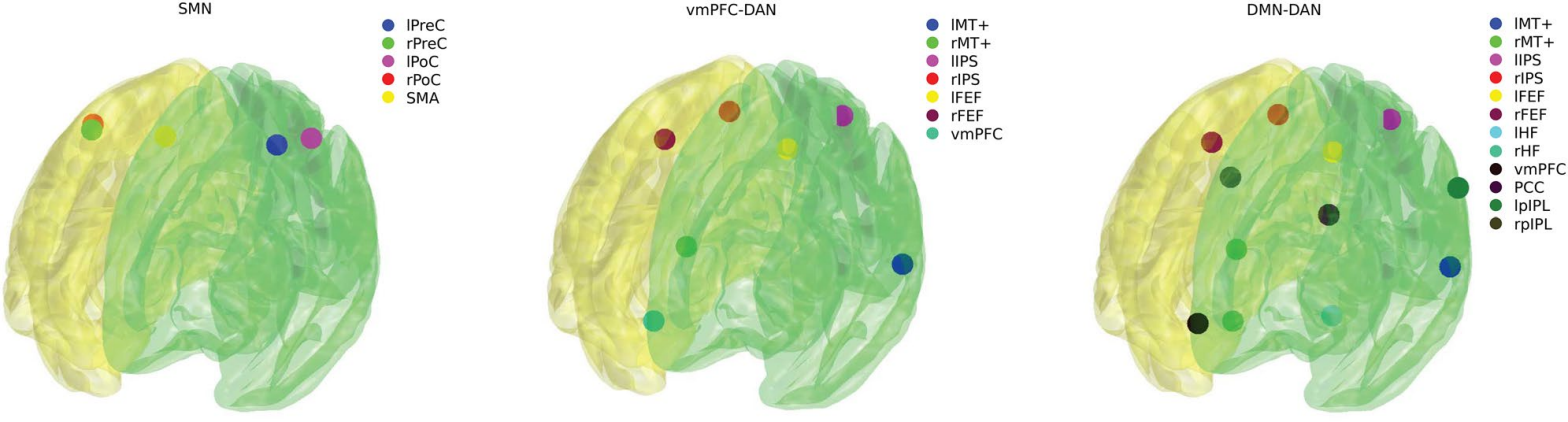
Representative ROIs of the *SMN* sensorimotor network, *vmPFC–DAN* ventromedial prefrontal cortex–dorsal attention network, and *DMN–DAN* default mode network–dorsal attention network.

Resting-state fMRI data were preprocessed using the SPM 12 toolbox and Matlab 2014b. Preprocessing started with realignment and unwarping. Next, cardiac and respiratory signals were regressed out using RETROICOR^59^. Then, normalization into standard anatomical space (MNI) and spatial smoothing with 5 mm FWHM was performed. The level of motion was thoroughly checked in terms of frame-wise displacement (FD)^60^. No FD was higher than 3 mm and scans that displayed FD > 0.75 mm were scrubbed^60^. No more than 2.5% of subject scans were removed. Moreover, the six movement regressors (obtained during realignment and unwarping), FD, and extracted signals from white matter and cerebrospinal fluid were regressed out of the data in the subsequent analysis. Representative regions of interest (ROIs; spheres with 6 mm radius) of large-scale functional brain networks of interest (SMN, DAN) and a vmPFC ROI representing the aDMN were chosen (see Fig. 2), together with the whole DMN, VN and FPCN for exploratory purposes based on a literature review^26^. MNI coordinates for each ROI and network are listed in the “Supplementary Materials” (Table S2). Mean ROI signals were extracted and a correlation matrix was calculated for each subject. Pearson’s correlation coefficients were converted to z values using Fisher’s r-to-z transformation. The connectivity within the SMN and between the DAN–aDMN were calculated as the average of z values within the network ROI pairs^61^.

### Graph theory

The whole brain (except the cerebellum) was parcelled into 90 regions of interest (ROIs) according to the AAL atlas^62^. These ROIs were intersected with previously calculated masks to ensure high signal quality in every subject. ROIs that contained more than 50% of signal dropouts in more than 10% of subjects were removed (4 in total). The time-series of each ROI was averaged and cross-correlated using Pearson’s correlation coefficient to form a 86 × 86 correlation matrix for each subject. Pearson’s correlation coefficients were converted into z-values using Fisher’s r-to-z transformation. Weighted networks were analysed so that the useful information about the strength of a particular connection was preserved. Negative correlations were replaced with zeros. Finally, global efficiency and modularity were computed using the Brain Connectivity toolbox^63^.

### Analyses of the CR effects on DI-induced changes in rs-FC

Demographics between the two study groups were compared using t-tests for continuous variables and chi-square tests for categorical variables. The CR was represented by years of education^19^; the program variable had two dimensions—dance intervention (DI) or control life as usual (LAU); changes in the outcome variables of interest were computed as timepoint_2_ (a follow-up visit after 6 months)—timepoint_1_ (baseline).

As discussed briefly, we also conducted one-tailed Pearson correlations between baseline and follow-up change of behavioral and rs-FC outcomes, and with education independently of the intervention. This was to test the association of behavioral outcomes and the rs-FC changes of interest, as well as their relationship with years of education.

In the moderation analyses, the effect of CR (moderator variable) was tested on the relationship between the program (independent variable) and change in functional outcomes of interest. The moderations were performed on 68 subjects who had fMRI data of sufficient quality (36 DI and 32 LAU) to assess whether and to what degree the relationship between DI and rs-FC changes within the SMN and between the DAN–aDMN depend on CR levels. Moderation was selected because it enables inferring the effect of CR, program, and their interaction (CR*program), as well as modeling the DI effect on different levels of the CR moderator. This approach simplifies the interpretation of results when such effects are present based on the zone of significance without the necessity of subsequent sets of single main effect tests. The significant moderations are plotted using the CRAN R package v 1.1.0. All data were normally distributed.

All statistical analyses were performed using IBM SPSS Statistics 27. For moderation analyses, we used the PROCESS macro for SPSS v 3.4. Finally, the Šidák-Dunn correction was set for each dataset independently in the following manner: 1 − (1 − α)^½^ = 0.025 for the two moderation models; the correlation analyses were exploratory and uncorrected.

## Supplementary Information

Supplementary Tables.

## Data Availability

The datasets analyzed during the current study are available from the corresponding author on reasonable request.
