# Supplementary material for: Impact of cognitive reserve on dance intervention-induced changes in brain plasticity
Source: Sci Rep. 2021 Sep 17;11:18527. doi: 10.1038/s41598-021-97323-2 (PMC8448766; doi:10.1038/s41598-021-97323-2)
Supplement: Supplementary file 1 — Supplementary Tables. [file 41598_2021_97323_MOESM1_ESM.docx]

**Supplementary materials**

| **Domains** | **Test/subtest** |
| --- | --- |
| Global cognition | Montreal Cognitive Assessment (Nasreddine et al., 2005) |
| Memory | Taylor figure test/ recall 3 min after copy (Taylor, 1969) |
|  | Taylor figure test/ recall 30 min after copy (Taylor, 1969) |
|  | WMS III/ Logical memory immediate recall (Wechsler, 1997) |
|  | WMS III/ Logical memory recall after 30 min (Wechsler, 1997) |
| Attention | WAIS III/ Symbol search (Wechsler, 1997) |
|  | WAIS III/ Digit span (Wechsler, 1997) |
| Executive function | Tower of Hanoi/ 3 discs (Humes et al., 1997) |
|  | Tower of Hanoi/ 4 discs (Humes et al., 1997) |
|  | Five-Point Test (Tucha et al., 2012) |
| Visuospatial function | Judgement of Line Orientation test (Benton et al., 1994) |
|  | Taylor figure/ copy (Taylor, 1969) |
| Self-dependence | Bristol Activities of Daily Living Scale (Bucks et al., 1996) |
| Depression | Beck Depression Inventory II (Beck et al., 1996) |

**Table S1***.* Neuropsychological assessment. Tests and cognitive domains.

| **Networks** | **Seeds** | **Coordinates** |
| --- | --- | --- |
| Sensorimotor network | lPreC | (-41, -4, 54) |
|  | rPreC | (42, -13, 53) |
|  | lPoC | (-45, -26, 54) |
|  | rPoC | (49, -27, 53) |
|  | SMA | (6, -5, 54) |
| Dorsal attention network | lMT+ | (-45, -69, -2) |
|  | rMT+ | (50, -69, -3) |
|  | lIPS | (-27, -52, 57) |
|  | rIPS | (24, -56, 55) |
|  | lFEF | (-25, -8, 50) |
|  | rFEF | (27, -8, 50) |
| Default mode network | vmPFC | (0, 51, -7) |
|  | lFH | (-21, -15, -14) |
|  | rHF | (24, -19, -21) |
|  | PCC | (1, -55, 17) |
|  | lpIPL | (-47, -71, 29) |
|  | rpIPL | (50, -64, 27) |
| Visual network | lCal | (-8, -72, 4) |
|  | rCal | (16, -67, 5) |
|  | lCS | (-5, -96, 12) |
|  | rCS | (18, -96, 12) |
|  | lLO | (-23, -89, 12) |
|  | rLO | (37, -85, 13) |
| Fronto-parietal control network | laPFC | (-36, 57, 9) |
|  | raPFC | (34, 52, 10) |
|  | ACC | (3, 31, 27) |
|  | laIPL | (-52, -49, 47) |
|  | raIPL | (52, -46, 46) |
|  | ldlPFC | (-50, 20, 34) |
|  | rdlPFC | (46, 14, 43) |
|  | lINS | (-31, 21, -1) |
|  | rINS | (31, 22, -2) |

**Table S2***.* MNI coordinates of regions of interest within the predefined networks according to Gao and Lin (2012). *Note*: *aPFC*, anterior prefrontal cortex; *dlPFC*, dorsal lateral prefrontal cortex; *ACC*, anterior cingulate cortex; *INS*, insula; *aIPL*, anterior inferior parietal lobule; *IPS*, bilateral intra-parietal sulcus; *FEF*, frontal eye field; *MTþ*, middle temporal area; *PCC*, posterior cingulate cortex; *MPFC*, medial prefrontal cortex; *pIPL*, bilateral posterior inferior parietal lobule; *HF*, hippocampus formation; *PreC*, precentral gyrus; *PoC*, postcentral gyrus; *SMA*, supplementary motor area; *Cal*, bilateral calcarine; *CS*, cuneus; *LO*, lateral occipital.

|  | **Variables at baseline** | | | | | **Change of variables (time_2_-time_1_)** | | | | |
| --- | --- | --- | --- | --- | --- | --- | --- | --- | --- | --- |
|  | **DI = 9** | **LAU = 12** | ***U*** |  | ***p*** | **DI** | **LAU** | ***U*** |  | ***p*** |
|  | **M ± SD** | **M ± SD** |  |  |  | **M ± SD** | **M ± SD** |  |  |  |
| Age | 70.39 ± 6.30 | 69.66 ± 6.48 | 48 | -.43 | .70 | - | - | - | - | - |
| Edu. | 13.89 ± 1.45 | 14.17 ± 3.04 | 46.5 | -.55 | .60 | - | - | - | - | - |
| MoCA | *****  **24 ± 3** | 25.17 ± 3.88 | 46.5 | -.54 | .60 | ******  **1.56 ± 1.51** | -.33 ± 3.03 | 32.5 | -1.55 | .13 |
| FPT | 23.11 ± 10.43 | 28.33 ± 8.82 | 38 | -1.14 | .28 | 3.11 ± 6.79 | -.33 ± 4.19 | 36.5 | -1.25 | .22 |
| 8UG | 5.31 ± .98 | 5.43 ± 1.29 | 52.5 | -.11 | .92 | -.51 ± .92 | -.03 ± 84 | 37 | -1.21 | .25 |
| 30CS | 14.33 ± 3.46 | 17.17 ± 6.49 | 41.5 | -.89 | .38 | 1.22 ± 3.19 | .67 ± 1.56 | 36.5 | -1.26 | .22 |
| SMN | .60 ± .12 | .60 ± .13 | 51 | -.21 | .86 | -.01 ± .23 | -.01 ± .19 | 53 | -.07 | .97 |
| DAN-aDMN | .05 ± .07 | .13 ± .11 | 28 | -.85 | .07 | .05 ± .09 | -.04 ± .10 | 27 | -1.92 | .06 |

**Table S3a.** Baseline and follow-up demographic comparison of MCI subjects in two experimental groups based on the Mann-Whitney U tests. *DI* – Dance-intervention group; *LAU* – Life-as-usual group; *Age* and *Education* in years; *MoCA* – Montreal Cognitive Assessment score; *FPT* – Five-Point Test score; *8UG* - 8-Foot Up-and-Go score in seconds; *30SC* – 30-Second Chair Stand test score; *SMN* – internetwork connectivity of the sensorimotor network; *DAN-aDMN* – connectivity between the dorsal attention network and the anterior default mode network; *M* – mean; *SD* – standard deviation. Bold values: significant differences between the DI_HC_ and the DI_MCI_ in the MoCA baseline* (U = 10, p <.001), the SMN (U = 58, p = .019); and in the MoCA change** (U = 35.5, p = .001).

|  | **Variables at baseline** | | | | | **Change of variables (time_2_-time_1_)** | | | | |
| --- | --- | --- | --- | --- | --- | --- | --- | --- | --- | --- |
|  | **DI = 27** | **LAU = 20** | ***U*** | ***Z*** | ***p*** | **DI** | **LAU** | ***U*** | ***Z*** | ***p*** |
|  | **M ± SD** | **M ± SD** |  |  |  | **M ± SD** | **M ± SD** |  |  |  |
| Age | 68.86 ± 5.24 | 68.65 ± 5.96 | 256 | -.30 | .76 | - | - | - | - | - |
| Edu. | 15.07 ±2.48 | 15.55 ± 2.96 | 243.5 | -.77 | .44 | - | - | - | - | - |
| MoCA | *****  **28.52 ± 1.45** | 26.20 ± 2.24 | 98.5 | -3.76 | .001 | ******  **-.89 ± 1.74** | 1.3 ± 2.60 | 137.5 | -2.89 | .004 |
| FPT | 30.37 ± 5.91 | 33.65 ± 9.30 | 201 | -1.49 | .14 | 4.59 ± 6.32 | .30 ± 8.89 | 195 | -1.62 | .11 |
| 8UG | 5.25 ± 1.39 | 5.17 ± 1.56 | 234 | -.58 | .56 | -.37 ± .84 | .62 ± 1.67 | 151 | -2.21 | .03 |
| 30CS | 15.77 ± 3.15 | 17.05 ± 4.5 | 221 | -.87 | .38 | 1.81 ± 2.70 | -.47 ± 2.82 | 127 | -2.78 | .005 |
| SMN | .52 ± .11 | .57 ± .21 | 246 | -.52 | .61 | .06 ± .14 | -.01 ± .13 | 177 | -2 | .045 |
| DAN-aDMN | .07 ± .09 | .06 ± .13 | 266 | -.09 | .93 | .02 ± .10 | -.03 ± .12 | 197 | -1.57 | .12 |

**Table S3b.** Baseline and follow-up demographic comparison of healthy subjects in two experimental groups based on the Mann-Whitney *U* tests. *Z*- Z-score; *DI* – Dance-intervention group; *LAU* – Life-as-usual group; *Age* and *Edu.* in years; *MoCA* – Montreal Cognitive Assessment score; *FPT* – Five-Point Test score; *8UG* - 8-Foot Up-and-Go score in seconds; *30SC* – 30-Second Chair Stand test score; *SMN* – internetwork connectivity of the sensorimotor network; *DAN-aDMN* – connectivity between the dorsal attention network and the anterior default mode network; *M* – mean; *SD* – standard deviation.

| **CR** | 1 |  |  |  |  |  |  |  |  |  |  |
| --- | --- | --- | --- | --- | --- | --- | --- | --- | --- | --- | --- |
| **MoCA** | **.22^*^** | 1 |  |  |  |  |  |  |  |  |  |
| **LM short recall** | **.23^*^** | .45^**^ | 1 |  |  |  |  |  |  |  |  |
| **LM delayed recall** | **.15** | .49^**^ | .86^**^ | 1 |  |  |  |  |  |  |  |
| **TF short recall** | **.16** | .28^**^ | .40^**^ | .48^**^ | 1 |  |  |  |  |  |  |
| **TF delayed recall** | **.16** | .33^**^ | .35^**^ | .46^**^ | .93^**^ | 1 |  |  |  |  |  |
| **Symbol search** | **.36^**^** | .37^**^ | .33^**^ | .28^**^ | .39^**^ | .35^**^ | 1 |  |  |  |  |
| **Digit span** | **.39^**^** | .48^**^ | .25^**^ | .25^**^ | .24^**^ | .28^**^ | .31^**^ | 1 |  |  |  |
| **TOH movements** | .14 | .38^**^ | .27^**^ | .40^**^ | .35^**^ | .33^**^ | .24^**^ | .12 | 1 |  |  |
| **TOH time** | **-.24^**^** | -.32^**^ | -.36^**^ | -.41^**^ | -.26^**^ | -.22^*^ | -.32^**^ | -.21^*^ | -.73^**^ | 1 |  |
| **JLO** | **.30^**^** | .23^*^ | .01 | .06 | .12 | .15 | .12 | .19^*^ | .09 | -.03 | 1 |
|  | **CR** | **MoCA** | **LM short recall** | **LM delayed recall** | **TF short recall** | **TF delayed recall** | **Symbol search** | **Digit span** | **TOH movements** | **TOH time** | **JLO** |

**Table S4a.** Baseline correlations of tests administered in the study and CR, values in bold (tests with CR) are significant after adjusting for age and sex. **Correlation is significant at the 0.01 level (1-tailed). *Correlation is significant at the 0.05 level (1-tailed). *Note*: *CR*, cognitive reserve; *LM*, Logical Memory; *TF*, Taylor Figure; *TOH*, Tower of Hanoi; *JLO*, Judgement of Line Orientation

| **CR** | 1 |  |  |  |  |  |  |  |  |  |  |
| --- | --- | --- | --- | --- | --- | --- | --- | --- | --- | --- | --- |
| **Δ MoCA** | .22^*^ | 1 |  |  |  |  |  |  |  |  |  |
| **Δ LM short recall** | .13 | .25^**^ | 1 |  |  |  |  |  |  |  |  |
| **Δ LM delayed recall** | .21^*^ | .32^**^ | .56^**^ | 1 |  |  |  |  |  |  |  |
| **Δ TF short recall** | .02 | .20^*^ | .10 | .11 | 1 |  |  |  |  |  |  |
| **Δ TF delayed recall** | -.05 | .16 | .07 | .16 | .77^**^ | 1 |  |  |  |  |  |
| **Δ Symbol search** | -.01 | -.11 | -.30^**^ | -.09 | .07 | .07 | 1 |  |  |  |  |
| **Δ Digit span** | **-.14** | .01 | -.08 | .08 | .09 | .05 | .02 | 1 |  |  |  |
| **Δ TOH movements** | **.12** | .12 | -.02 | .17^*^ | -.02 | .03 | .07 | .02 | 1 |  |  |
| **Δ TOH time** | .07 | .04 | .00 | .17^*^ | -.02 | .03 | .07 | .02 | .76^**^ | 1 |  |
| **Δ JLO** | -.30^**^ | -.05 | -.20^*^ | -.11 | -.13 | -.05 | -.01 | -.03 | -.10 | -.04 | 1 |
|  | **CR** | **Δ MoCA** | **Δ LM short recall** | **Δ LM delayed recall** | **Δ TF short recall** | **Δ TF delayed recall** | **Δ Symbol search** | **Δ Digit span** | **Δ TOH movements** | **Δ TOH time** | **Δ JLO** |

**Table S4b.** Correlations between change in tests and CR, values in bold (tests with CR) are significant after adjusting for age and sex **Correlation is significant at the 0.01 level (1-tailed). *Correlation is significant at the 0.05 level (1-tailed). *Note*: ∆ - change time_2_ – time_1_; *CR*, cognitive reserve; *LM*, Logical Memory; *TF*, Taylor Figure; *TOH*, Tower of Hanoi; *JLO*, Judgement of Line Orientation

| **Model terms** | **B [95% CI]** | **SE B (HC4)** | **t** | **p** |
| --- | --- | --- | --- | --- |
| **DAN-DMN change** | | | | |
| Constant | -.131 [-.367, .105] | .118 | -1.111 | .271 |
| Program | .045 [.004, .086] | .021 | 2.170 | .034 |
| CR (centered) | .003 [-.009, 015] | .006 | .527 | .600 |
| Moderator CR*program | .001 [-.016, .019] | .009 | .145 | .885 |
| Age | .002 [-.002, .005] | .002 | 1.042 | .302 |
| Sex | -.008 [-.067, .050] | .029 | -.286 | .775 |
| **FPCN-DMN change** | | | | |
| Constant | .070 [-.191, .332] | .131 | .537 | .593 |
| Program | -.004 [-.042, .033] | .019 | -.229 | .820 |
| CR (centered) | .002 [-.009, 012] | .005 | .328 | .744 |
| Moderator CR*program | .014 [-.001, .029] | .007 | 1.915 | .060 |
| Age | -.001 [-.005, .003] | .002 | -.476 | .636 |
| Sex | -.004 [-.053, .045] | .025 | -.166 | .869 |
| **VN-DMN change** | | | | |
| Constant | -.068 [-.335, .199] | .134 | -.510 | .612 |
| Program | .041 [-.012, .094] | .027 | 1.535 | .130 |
| CR (centered) | .000 [-.016, .016] | .008 | .002 | .998 |
| Moderator CR*program | .004 [-.019, .026] | .011 | .320 | .750 |
| Age | .000 [-.003, .004] | .002 | .223 | .824 |
| Sex | .007 [-.058, .071] | .032 | .203 | .839 |
| **SMN-DMN change** |  |  |  |  |
| Constant | -.169 [-.449, .110] | .140 | -1.212 | .230 |
| Program | .043 [-.002, .087] | .022 | 1.923 | .059 |
| CR (centered) | .001 [-.011, .012] | .006 | .088 | .930 |
| Moderator CR*program | .000 [-.016, .017] | .008 | .043 | .966 |
| Age | .002 [-.001, 006] | .002 | 1.268 | .210 |
| Sex | -.036 [-.102, .031] | .033 | -1.073 | .287 |

**Table S5***.* Linear model of predictors of rsFC change between the DAN-DMN (R^2^ = .086; F(5,62) = 1.255; p = .295), FPCN-DMN (R^2^ = .129; F(5,62) = 2.626; p = .032), VN-DMN connectivity (R^2^ = .050; F(5,62) = .594; p = .704), ), SMN-DMN connectivity (R^2^ = .080; F(5,62) = 1.688; p = .151); Results are unstandardized beta coefficients with 95% confidence intervals from moderation models estimating the association of program, CR, and their interaction with rs-FC change and accounting for age and sex as covariates. Note: only continuous variables that contributed to the outcomes were centered. Cribari-Neto model was used for standard error of variance and F-statistics. Variable program coded as *LAU*: 0, *DI*: 1; *DMN* – default mode network; *DAN* - dorsal attention network; *FPCN* – fronto-parietal control network; *VN* – visual network; *SMN* – sensorimotor network; *CR*, cognitive reserve.

| **Model terms** | **B [95% CI]** | **SE B (HC4)** | **t** | **p** |
| --- | --- | --- | --- | --- |
| **Global modularity change** | | | | |
| Constant | .000 [-.126, .126] | .063 | .002 | .999 |
| Program | -.016 [-.039, .007] | .011 | -1.407 | .164 |
| CR | .002 [-.003, .008] | .003 | .862 | .392 |
| Moderator CR*program | .003 [-.008, .014] | .005 | .539 | .592 |
| Age | .000 [-.002, .002] | .001 | -.019 | .985 |
| Sex | .014 [-.012, .041] | .013 | 1.096 | .277 |
| **Global efficiency change** | | | | |
| Constant | -.054 [-.287, .180] | .117 | -.459 | .648 |
| Program | .032 [.003, .061] | .014 | 2.200 | .032 |
| CR | .000 [-.007, .007] | .003 | .039 | .969 |
| Moderator CR*program | .011 [-.002, .025] | .007 | 1.632 | .108 |
| Age | .001 [-.003, .004] | .002 | .424 | .673 |
| Sex | -.003 [-.044, .038] | .021 | -.163 | .871 |

**Table S6.** Linear model of predictors of global modularity (R^2^ = .056; F(5,62) = .695; p = .629) and efficiency (R^2^ = .132; F(5,62) = 1.878; p = .111); Results are unstandardized beta coefficients with 95% confidence intervals from moderation models estimating the association of program, CR, and their interaction with rs-FC change and accounting for age and sex as covariates. Note: only continuous variables that contributed to the outcomes were centered. Cribari-Neto model was used for standard error of variance and F-statistics. Variable program coded as *LAU*: 0, *DI*: 1; *CR*, cognitive reserve.
